# Impact of geometric factors and shear stress on endothelial function: Hemodynamic insights from human cerebral arterial bifurcations

**DOI:** 10.14814/phy2.70970

**Published:** 2026-06-07

**Authors:** Ilias Miltiadis, Oleg Zenin, Edgar Kafarov, Mahsa Alavi, Nadezhda Stashevskaya, Pavel Burko

**Affiliations:** ^1^ Department of Biomedicine, Neuroscience and Advanced Diagnostics (BiND) University of Palermo Palermo Italy; ^2^ Penza State University Penza Russia; ^3^ A.A. Kadyrov Chechen State University Grozny Russia; ^4^ RUDN University Moscow Russia; ^5^ Synergy University Moscow Russia

**Keywords:** arterial bifurcation, cerebral hemodynamics, endothelial cell activation potential (ECAP), mechanobiology, vascular inflammation, wall shear stress

## Abstract

Vascular inflammation is not a random biological event but rather a process that is strictly regulated by physical forces. While biochemical mediators are well characterized, the local physical mechanisms that predispose specific cerebral vascular sites to inflammation, specifically the geometry of arterial bifurcations, remain underappreciated. This study aims to determine how distinct arterial bifurcation geometries physically regulate the local inflammatory microenvironment by modulating wall shear stress (WSS), relative residence time (RRT), and endothelial cell activation potential (ECAP). We performed a high‐resolution morphometric analysis of 1512 arterial bifurcations. They were categorized based on parent–daughter diameter relationships: open‐shaped (87.4%), neutral‐shaped (10.4%), and closed‐shaped (2.2%). The open‐shaped configuration acts as a low‐shear trap, exhibiting the highest median RRT (1.68 s) and ECAP (0.092). The closed‐shaped configuration concentrates mechanical energy, subjecting the endothelium to significantly elevated high median WSS (7 Pa) and peak pressures (17.16 kPa). Cerebral arterial bifurcations create distinct hemodynamic microenvironments that govern local vascular vulnerability. The prevalent open‐shaped geometry minimizes energy loss at the cost of a high residence time, physically priming the endothelium for stagnation‐induced inflammation. In contrast, closed‐shaped geometries protect downstream microvessels by dissipating energy but sacrifice the local endothelium to high‐shear mechanical stress.

## 1 INTRODUCTION

The regulation of cerebral vascular health involves a sophisticated interplay among structural anatomy, fluid dynamics, and cellular biology. While inflammation has traditionally been viewed through a biochemical lens governed by systemic risk factors, a paradigm shift has occurred in recent years (Feng et al., 2025). Hemodynamic forces are active regulators of the vascular phenotype, with the cerebral endothelium functioning as a mechanosensory organ that continuously gauges the physical environment (Fan et al., 2025; Ramses & Agu, 2025). This “hemodynamic hypothesis” posits that local flow disturbances—specifically, wall shear stress (WSS), the oscillatory shear index (OSI), and the relative residence time (RRT)—are the primary determinants of the focal distribution of vascular pathology, directing where inflammation occurs within the complex geometry of the intracranial arterial tree.

The intracranial arterial system comprises four major vessels—the anterior, middle, and posterior cerebral arteries and the basilar artery—which form an interconnected network through the Circle of Willis. In addition, the vasculature branches into progressively smaller vessels whose geometry can be described via mathematical models of bifurcation. These arterial bifurcations function as structural “LEGO blocks” of the cerebral vasculature, determining local flow dynamics and shaping downstream hemodynamics. However, despite their central role, bifurcation‐level configurations remain understudied compared with the well‐characterized large proximal segments.

Atherosclerosis in the cerebral circulation is highly focal and develops predominantly at bifurcations and along outer arterial curves. At these locations, endothelial cells experience low time‐averaged wall shear stress (TAWSS) and a high OSI, which promote endothelial activation, inflammation, and lipid accumulation (Ahmadpour‐B et al., 2021; Ghaffari et al., 2018). High‐resolution vessel wall MRI combined with computational fluid dynamics (CFD) has shown that plaque burden in non‐stenotic arteries can itself be an independent risk factor for stroke. Even when luminal narrowing is <50%, plaques in regions of high endothelial cell activation potential (ECAP) or high RRT may be unstable (Sun et al., 2022). The plaque burden and enhancement ratio (a marker of active inflammation) are statistically significant predictors of recurrent stroke (HR = 3.15 for plaque burden, *p* = 0.009) (Lv et al., 2024). These observations challenge the traditional focus on high‐grade stenosis and emphasize the role of local hemodynamics at smaller‐scale vascular structures.

The lesions predominantly cluster at arterial bifurcations, branch points, and regions of high curvature—sites characteristically exposed to “disturbed flow” (Jin & Fu, 2019), which is hemodynamically defined by a low TAWSS, flow separation, recirculation, and a high degree of directional oscillation. In contrast, straight arterial segments experience laminar, unidirectional flow with high physiological shear stress, which exerts an atheroprotective influence by promoting the expression of anti‐inflammatory and antioxidant genes. Recent research has identified the Piezo1 ion channel as a critical gatekeeper in this process (Lan et al., 2024). Piezo1 senses the membrane tension generated by shear stress and gates Ca^2+^ influx. Under oscillatory conditions, this calcium signal activates calmodulin‐dependent protein kinase II (CaMKII) and the transcriptional coactivator YAP, which cooperate with nuclear factor‐kappa B (NF‐κB) to drive the expression of adhesion molecules such as VCAM‐1 and ICAM‐1 (Hou et al., 2025; Lan et al., 2024). Furthermore, disturbed flow has been shown to activate the NLRP3 inflammasome, a multiprotein complex that links physical stress to innate immunity. The hemodynamic environment provides both the “priming” signal (via NF‐κB) and the “activation” signal (via Piezo1‐mediated K^+^ efflux and mitochondrial dysfunction) required for the release of high‐potency cytokines such as IL‐1β (Grebe et al., 2018).

These inflammatory forces are not distributed randomly; rather, they are strictly dictated by vascular geometry. The cerebral arterial system is constructed from thousands of bifurcations, which serve as the fundamental architectural units of the network. Despite the critical role of these geometries, most research has focused on large segments of the Circle of Willis or patient‐specific aneurysm models. A critical gap remains in understanding how fundamental morphological variations in arterial bifurcations physically regulate the local inflammatory microenvironment.

The aim of this study is to address this gap by analyzing the hemodynamic parameters associated with different bifurcation‐level configurations across the arterial network.

## 2 MATERIALS AND METHODS

### 2.1 Study design

This study used high‐resolution corrosion casts of human cerebral arterial vasculature to characterize hemodynamic behavior across different bifurcation configurations. The study utilized corrosive casts of the arterial cerebral vasculature from 5 men, aged 46–57 years. Forty‐eight corrosion casts were obtained from five adult human brains. The inclusion criteria were as follows: age >18 years, death from nonvascular accidental causes, and absence of visible arterial deformation. The exclusion criteria included vascular disease, traumatic organ damage, or casting defects.

### 2.2 Corrosion casting

Corrosion casting was performed according to established protocols (Kafarov et al., 2023). After the autopsy, the arterial vasculature of the brain was washed with saline for 40–45 min at a constant pressure of 100 mm Hg and then placed in a vessel filled with saline to prevent deformation of the organ due to gravity. A radiopaque mixture was injected into the arterial vasculature under a constant pressure of 100 mm Hg. The mixture contains a polymer, “Protacryl M” (Stoma; Ukraine), which consists of dry and liquid components, a radiopaque agent, barium sulfate, and a universal color component (Kafarov et al., 2021). The ratio of the mixture in mass % was as follows: “Protacryl M” powder—50%; “Protacryl M” liquid—20%; barium sulfate—20%; and dye—10%. The container with the brain was incubated at 36°C for 24 h. Then, the brain was dipped into a solution of full‐strength alkali for 3–4 days in a thermal chamber. Tap water was used to rinse the corrosive cast. The minimum diameter of the cast vascular segments was 0.05 mm.

### 2.3 Analysis of the vascular casts

The parameters of each vascular segment of the corrosion casts were measured with sequential step‐by‐step defragmentation of the cast: *D*—diameter of the vascular segment in its central part (in the middle of the distance between the nearest branches) (mm), *L*—length of the vascular segment (mm)—the shortest distance between two branching points. Measurements were made via calipers with a mechanical and electronic scale with a measurement accuracy of up to 0.01 mm. The minimum diameter of the vascular segment casts measured was 0.1 mm.

**FIGURE 1:**
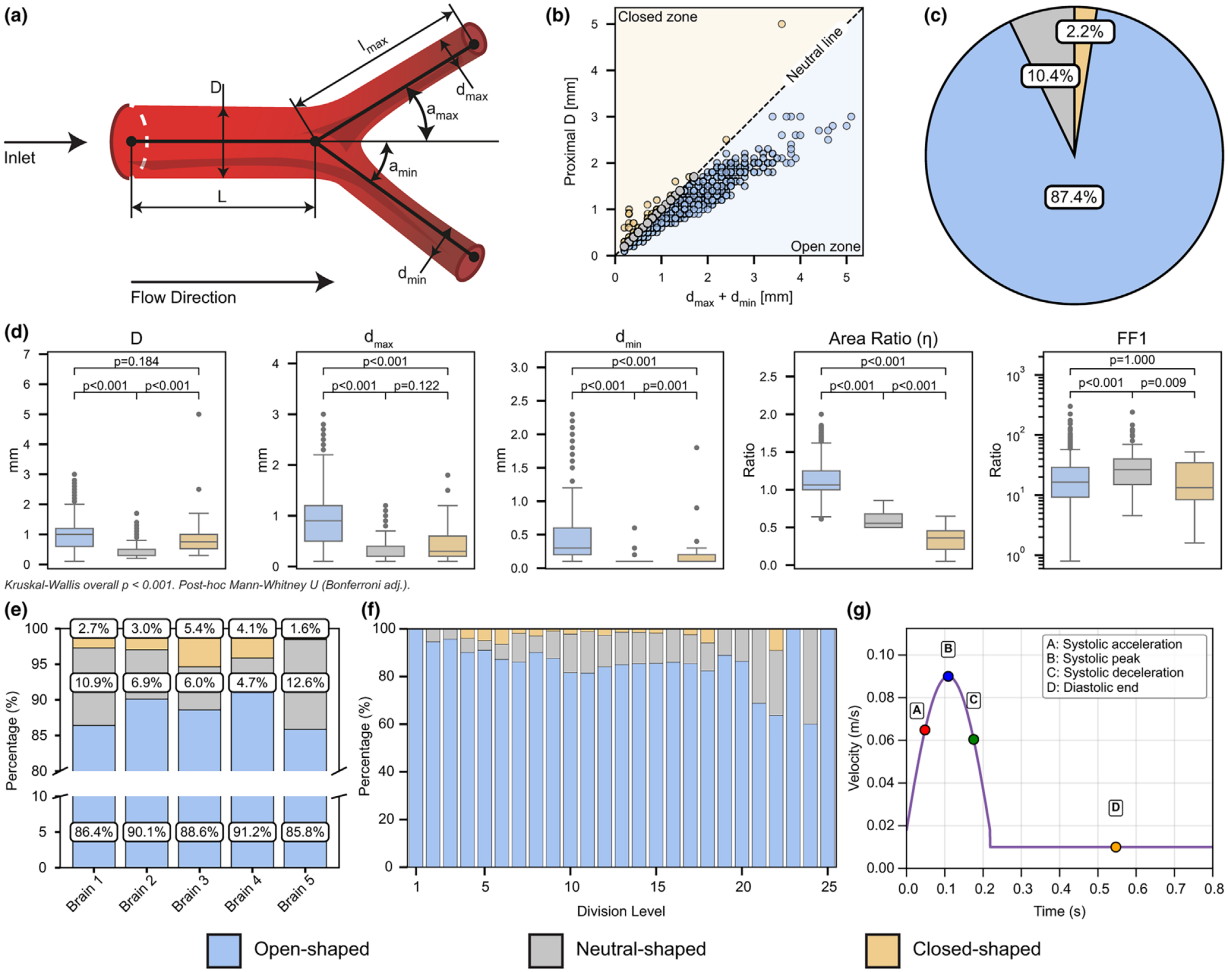
(a) Schematic of the cerebral arterial bifurcation, the fundamental architectural unit of the vascular tree. (b) Scatter plot of D versus the sum of the daughter diameters ($d_{\max}+d_{\min}$), demonstrating the mathematical boundaries used for classifying the bifurcations into open, neutral, and closed configurations. (c) Overall prevalence of configuration phenotypes across the total analyzed sample. (d) Boxplots detailing the distribution of key morphometric parameters—*D* (mm), $d_{\max}$ (mm), $d_{\min}$ (mm), asymmetry ratio (η), and form factor (FF1)—across the distinct configurations, complete with post hoc statistical analysis. (e) Proportional distribution of the bifurcation configurations across each of the five individual brains. (f) Distribution of the bifurcation configurations across successive vascular division levels. (g) The pulsatile inlet velocity profile applied during the CFD simulations.

The cerebral arterial vasculature (CAV) is considered a structure consisting of interconnected bifurcations—structural elements of the vasculature (Figure 1a). To describe such a structure on the basis of morphometric data, the following parameters were determined (Zenin et al., 2021):

1. *i*—the ordinal number of the division level of the newly formed series of vascular segments;
2. *D*—diameter of the proximal segment, located at the initial level of division;
3. *d*_max_—diameter of the distal branch with the larger value;
4. *d*_min_—diameter of the distal branch with the smaller value;
5. *L*—segment length, the distance between the nearest branching points;
6. *l*_max_—length of the distal segment associated with a larger diameter branch;
7. *l*_min_—length of the distal segment associated with a smaller diameter branch;
8. *FF*_1_—length‐to‐radius ratio ($FF_{1}=\frac{L}{R}$);
9. 𝛾—asymmetry ratio from the equation: $\gamma ={\left(\frac{d_{\min}}{d_{\max}}\right)}^{2}$(Olufsen et al., 2000);
10. 𝜂—area ratio from equation: $\eta =\frac{\pi d_{\max}^{2}+\pi d_{\min}^{2}}{\pi D^{2}}$ (Uylings, 1977).

The bifurcations were divided into three configurations:

Open‐shaped (1)—the value of the inner diameter (D) of the maternal (proximal) arterial segment is less than the sum of the inner diameters ($d_{\max}$ and $d_{\min}$) of the daughter (distal) segments: $D<d_{\max}+d_{\min}$;

Neutral‐shaped (0)—the value of the inner diameter of the maternal (proximal) arterial segment is equal to the sum of the inner diameters of the daughter (distal) segments: $D=d_{\max}+d_{\min}$;

Closed‐shaped (2)—the value of the inner diameter of the maternal (proximal) arterial segment is greater than the sum of the inner diameters of the daughter (distal) segments: $D>d_{\max}+d_{\min}$.

To investigate configuration‐specific hemodynamics, three representative geometries—one for each bifurcation category—were selected from the larger morphometric dataset.

### 2.4 Hemodynamics modeling

CFD simulations were performed in ANSYS Fluent using nonstationary three‐dimensional flow. Vessel walls were modeled as rigid, a physiologically acceptable approximation for small intracranial arteries given minimal differences in WSS between rigid‐ and elastic‐wall simulations (Gharahi et al., 2016; Shibeshi & Collins, 2005).

The fluid dynamics were governed by the continuity equation and the Navier–Stokes equation for incompressible, unsteady laminar flow. To account for shear‐thinning behavior, blood was modeled as a non‐Newtonian fluid with a density of 1060 kg/m^3^ and a dynamic viscosity that varies according to the Carreau model (Gharahi et al., 2016; Siebert & Fodor, 2009). The PISO algorithm was used to couple velocity and pressure (Issa, 1986).

The geometric models for the bifurcations were constructed using theoretical branching angles derived from C. D. Murray's equations (Murray, 1926a).

Boundary conditions were defined as follows: A pulsatile velocity profile was applied at the inlet (Figure 1g). At the outlet, a static manometric pressure of 100 mm Hg (approximately 13.33 kPa) was applied, representing the average of healthy systolic (120 mm Hg) and diastolic (80 mm Hg) pressures. The governing equations are provided in Appendix A.

### 2.5 Hemodynamic parameter definitions

To characterize the complex intracranial hemodynamics across the three bifurcation configurations, this study evaluated time‐averaged hemodynamic parameters derived from the WSS. Specifically, we quantified TAWSS, OSI, RRT, and ECAP. All the metrics were computed from surface‐resolved CFD fields using standard definitions.

The WSS is defined as the tangential force per unit area exerted by the blood flow on the endothelial surface (Katritsis et al., 2007). The TAWSS represents the median magnitude of the WSS vector integrated over the cardiac cycle (He & Ku, 1996; Wentzel et al., 2012). The transverse WSS (transWSS) measures the multidirectional WSS on a surface over time (Peiffer et al., 2013). To assess the directional variation in the WSS vector relative to the primary flow direction, the OSI was used. This dimensionless parameter quantifies the cyclic departure of the WSS vector from its predominant direction (Ku et al., 1985). The ECAP index combines shear magnitude with directional oscillation, characterizing the susceptibility of the arterial wall to thrombosis. High ECAP values indicate regions of high endothelial vulnerability, typically where a high OSI coincides with a low TAWSS (Boniforti et al., 2022; Di Achille et al., 2014). The RRT is the residence time of blood particles near the vessel wall (Himburg et al., 2004). Elevated RRT indicates stagnation zones where particles remain for extended periods, increasing the risk of thrombus formation (Rayz et al., 2010).

### 2.6 Statistical analysis

Statistical analysis and data visualization were performed using Python (libraries: SciPy.stats and Matplotlib). Descriptive statistics are presented as medians (Me) with interquartile ranges (IQRs). Differences between the morphometric data of the three configurations (open, neutral, and closed‐shaped) were first evaluated using the Kruskal–Wallis H test. A *p* value of <0.05 was considered statistically significant. Because CFD simulations were performed on three representative bifurcation geometries, the goal was to characterize the configuration‐specific hemodynamic signatures. Therefore, no statistical tests were applied to nodal CFD data, and no nodewise comparisons were performed. The reported values reflect spatially averaged hemodynamic metrics within each representative bifurcation model. To account for the hierarchical structure of the dataset, Linear Mixed‐Effects Models (LMM) were subsequently applied to the core morphometric parameters ($D,d_{\max},d_{\min}$). The geometric configuration was modeled as a fixed effect, while the individual specimen was modeled as a random effect (group intercept) to control for intra‐subject correlation.

## 3 RESULTS

### 3.1 Cerebral arterial vasculature morphometry

The corrosion casting study included 1512 cerebral arterial bifurcations. The descriptive statistics reveal a pronounced departure from normality for the majority of variables (Table 1).

**TABLE 1:** Values of studied parameters of cerebral arterial bifurcations.

Parameter	Mean	Median	SD	IQR	Range	Min	Max	Shapiro‐W	*p* Value
*D* (mm)	0.913	0.9	0.508	0.7	4.9	0.1	5.0	0.933	<0.001
*d*_max_ (mm)	0.831	0.8	0.485	0.7	2.9	0.1	3.0	0.953	<0.001
*d*_min_ (mm)	0.396	0.3	0.352	0.4	2.2	0.1	2.3	0.789	<0.001
*L* (mm)	8.971	6.8	8.027	7.5	70.8	0.2	71.0	0.779	<0.001
*l*_max_ (mm)	9.326	7.0	8.228	7.925	70.5	0.5	71.0	0.76	<0.001
*l*_min_ (mm)	7.386	5.3	7.012	6.0	66.8	0.2	67.0	0.712	<0.001
FF1	23.539	17.045	22.586	20.0	299.2	0.8	300.0	0.705	<0.001
*γ*	0.333	0.25	0.312	0.439	0.996	0.004	1.0	0.854	<0.001
*η*	1.045	1.04	0.296	0.318	1.95	0.05	2.0	0.979	<0.001

The diameter of the proximal segment (D) exhibited a median of 0.900 mm (IQR = 0.700 mm), indicating a positively skewed distribution. The diameters of the distal branches were asymmetric; the larger branch ($d_{\max}$) had a median of 0.800 mm (IQR = 0.700 mm), while the smaller branch ($d_{\min}$) showed a lower median of 0.300 mm (IQR = 0.400 mm) and a higher degree of skewness. The segment length (L), defined as the distance between branching points, was highly variable with a median of 6.800 mm (IQR = 7.500 mm). The lengths of the distal segments also varied considerably, with the segment associated with $d_{\max}$ ($l_{\max}$) having a median length of 7.00 mm (IQR = 7.925 mm) and the segment associated with $d_{\min}$ ($l_{\min}$) being shorter, with a median of 5.300 mm (IQR = 6.00 mm).

The derived parameters further describe the vascular geometry. The length‐to‐radius ratio (FF) was extremely skewed, with a median of 17.045 (IQR = 20.00). The asymmetry ratio (γ), calculated as (${\frac{d_{\min}}{d_{\max}}}^{2}$), had a median of 0.25 (IQR = 0.439), confirming a general trend of asymmetric branching, the area ratio (η) had a median of 1.04 (IQR = 0.318).

The cerebral arterial bifurcations were categorized into three distinct morphological configurations on the basis of their diameter relationships: open‐shaped ($D<d_{\max}+d_{\min}$), neutral‐shaped ($D=d_{\max}+d_{\min}$), and closed‐shaped ($D>d_{\max}+d_{\min}$) (Figure 1b,c). Kruskal–Wallis tests revealed statistically significant differences (*p* < 0.001) across all measured parameters, confirming that these configurations represent fundamentally distinct geometric patterns (Table 2, Figure 1d). Because the bifurcations were derived from five individual specimens, a mixed‐effects modeling approach was utilized to confirm that the observed morphological differences were not driven by patient‐specific nesting. The Intraclass Correlation Coefficients indicated that subject‐specific variance accounted for 10.0%, 9.1%, and 3.8% of the variability in D, $d_{\max}$, and $d_{\min}$, respectively. After controlling this hierarchical random effect, the mixed linear models confirmed that the structural differences between the open, neutral, and closed configurations remained statistically significant across all primary diameters (p<0.05 for all comparisons). This confirms that these geometric phenotypes are distinctly conserved architectural features across the dataset, independent of the individual subject.

**TABLE 2:** Morphometric parameters of various configurations in cerebral vasculature.

Parameter Me (95% CI)	Open‐shaped	Neutral‐shaped	Closed‐shaped	Kruskal *p* value
*D* (mm)	1.00 (0.60; 1.20)	0.30 (0.30; 0.50)	0.75 (0.53; 1.00)	<0.001
*d*_max_ (mm)	0.90 (0.50; 1.20)	0.20 (0.20; 0.40)	0.30 (0.20; 0.60)	<0.001
*d*_min_ (mm)	0.30 (0.20; 0.60)	0.10 (0.10; 0.10)	0.10 (0.10; 0.20)	<0.001
*L* (mm)	7.00 (4.00; 12.00)	4.60 (2.80; 8.00)	4.00 (2.85; 12.30)	<0.001
*l*_max_ (mm)	7.30 (4.40; 12.00)	5.00 (3.50; 8.00)	4.50 (3.42; 9.20)	<0.001
*l*_min_ (mm)	5.70 (3.50; 10.00)	4.00 (2.00; 6.00)	4.00 (2.32; 5.52)	<0.001
FF1	16.47 (9.23; 28.89)	26.67 (15.00; 40.00)	13.33 (8.39; 34.57)	<0.001
*γ*	0.25 (0.07; 0.49)	0.25 (0.06; 1.00)	0.25 (0.11; 0.89)	0.078
*η*	1.06 (1.00; 1.25)	0.56 (0.50; 0.68)	0.36 (0.21; 0.46)	<0.001

The sensitivity analysis confirmed the robust presence of the closed‐shaped configuration independent of potential casting artifacts. Applying the strict 20% asymmetric expansion to the daughter vessels across the dataset (*n* = 1512), 22 of the original 34 closed‐shaped bifurcations maintained their defining $D>d_{\max}+d_{\min}$ geometry. This represents a 64.7% survival rate under extreme corrective conditions, maintaining a strict baseline prevalence of 1.46% within the total cerebral arterial network. To completely erase this geometric phenotype, the daughter vessels would require a differential shrinkage rate vastly exceeding the 20% maximums reported in polymer chemistry literature. Thus, while the casting process may marginally inflate the raw count of these bifurcations, the closed‐shaped configuration represents a genuine and resilient anatomical feature rather than a purely methodological artifact.

Open‐shaped bifurcations represent the most prevalent configuration in larger vessels. These bifurcations are characterized by the largest maternal segment diameter (D=1.0mm) and substantial daughter vessel dimensions ($d_{\max}=0.9\;\mathrm{mm}$, $d_{\min}=0.3\;\mathrm{mm}$). They exhibit the longest segment lengths (L=7.0mm, $l_{\max}=7.3\;\mathrm{mm}$, $l_{\min}=5.7\;\mathrm{mm}$) and the most balanced length‐to‐radius ratio (FF=16.47). The asymmetry ratio (γ=0.25) indicates consistent branching asymmetry, while the area ratio (η=1.06) demonstrates near‐perfect conservation of cross‐sectional area, suggesting hemodynamic efficiency with minimal energy loss at these junctions.

Neutral‐shaped bifurcations represent an intermediate morphology with the smallest maternal diameter (D=0.3mm) and substantially reduced daughter vessel dimensions ($d_{\max}=0.2\;\mathrm{mm}$, $d_{\min}=0.1\;\mathrm{mm}$). These bifurcations exhibit moderate segment lengths (L=4.6mm, $l_{\max}=5.0\;\mathrm{mm}$, $l_{\min}=4.0\;\mathrm{mm}$), but the highest length‐to‐radius ratio (FF=26.67), indicating relatively elongated, slender segments. The area ratio (η=0.56) reveals a significant reduction in total cross‐sectional area, potentially influencing flow dynamics and pressure distribution. The wider confidence interval for asymmetry ratio (γ=0.25, IQR 0.06; 1.00) suggests greater variability in branching symmetry within this group.

Closed‐shaped bifurcations demonstrate a constrictive pattern with an intermediate maternal diameter (D=0.75mm) but disproportionately small daughter vessels ($d_{\max}=0.3\;\mathrm{mm}$, $d_{\min}=0.1\;\mathrm{mm}$). These exhibit the shortest segment lengths (L=4.0mm) with considerable variability (IQR: 2.85; 12.3 mm) and the lowest length‐to‐radius ratio (FF=13.33), indicating relatively stubby, wide segments. The markedly reduced area ratio (η=0.36) likely creates significant flow resistance and energy dissipation points within the vascular network.

To ensure that the observed geometric phenotypes were not anomalies isolated to a single individual, the distribution of bifurcation types was analyzed per specimen. Across the five brains, the closed‐shaped configuration was consistently identified in every subject, representing between 1.5% and 5.4% of the analyzed bifurcations per brain (Figure 1e). The overall distribution of open, neutral, and closed geometries did not significantly differ between individuals, confirming that these configurations are conserved anatomical features across the cerebral arterial tree rather than patient‐specific variants.

This morphological diversity suggests specialized functional adaptations within different regions of the cerebral arterial tree, potentially related to flow regulation, pressure maintenance, or metabolic requirements of specific brain territories.

### 3.2 CFD simulations

**FIGURE 2:**
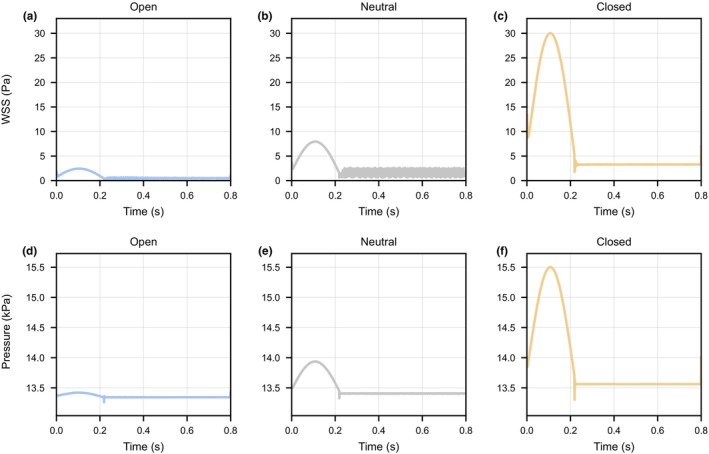
Temporal evolution of wall shear stress (WSS) (top row, a–c) and pressure (bottom row, d–f) throughout a single cardiac cycle for the three geometric configurations. The line graphs represent spatially averaged values across the entire bifurcation surface to illustrate global temporal trends. Absolute focal maximums (hotspots) at peak systole are significantly higher and are detailed in Table 4.

The hemodynamic characteristics of the open‐shaped, neutral‐shaped, and closed‐shaped bifurcation configurations were analyzed via CFD. Visualizations illustrating pressure and WSS dynamics throughout the cardiac cycle are available for the open‐shaped, neutral‐shaped, and closed‐shaped configurations in Figure 2. The detailed hemodynamic parameters are summarized in Table 3.

**TABLE 3:** Spatially averaged hemodynamic metrics for representative bifurcation models.

Parameter, Me (IQR)	Open‐shaped	Neutral‐shaped	Closed‐shaped
Pressure (kPa)	13.35 (13.34–13.36)	13.43 (13.38–13.48)	13.64 (13.48–13.73)
TAWSS (Pa)	0.813 (0.695–1.082)	2.396 (2.313–2.471)	7.416 (7.089–7.602)
transWSS (Pa)	0.008 (0.001–0.024)	0.004 (0.001–0.013)	0.0018 (0.001–0.012)
OSI	0.06 (0.001–0.1)	0.13 (0.001–0.14)	0.01 (0.001–0.01)
RRT (s)	1.68 (1.09–1.73)	0.57 (0.5–0.58)	0.13 (0.13–0.14)
ECAP	0.092 (0.0–0.145)	0.056 (0.0–0.058)	0.013 (0.013–0.016)

*Note*: Data represents median and interquartile range of values across surface mesh nodes within a single representative geometry for each configuration, not a population average.

The closed‐shaped configuration presented the highest median pressure and TAWSS. The median pressure values were 13.64 kPa for closed‐shaped, 13.43 kPa for neutral, and 13.35 kPa for open (Table 3, Figure 2d–f).

For the TAWSS, the median values were 0.81 Pa (open), 2.396 Pa (neutral), and 7.416 Pa (closed). The maximum TAWSS observed in the closed configuration reached 19.41 Pa.

The median RRT was highest in the open configuration (1.68 s), whereas the lowest RRT was found in the closed configuration (0.13 s). The open‐shaped configuration also presented the highest median value for ECAP (0.092). The median transWSS was highest in the open configuration (0.0078 Pa).

### 3.3 Hemodynamic parameters through the cardiac cycle

**FIGURE 3:**
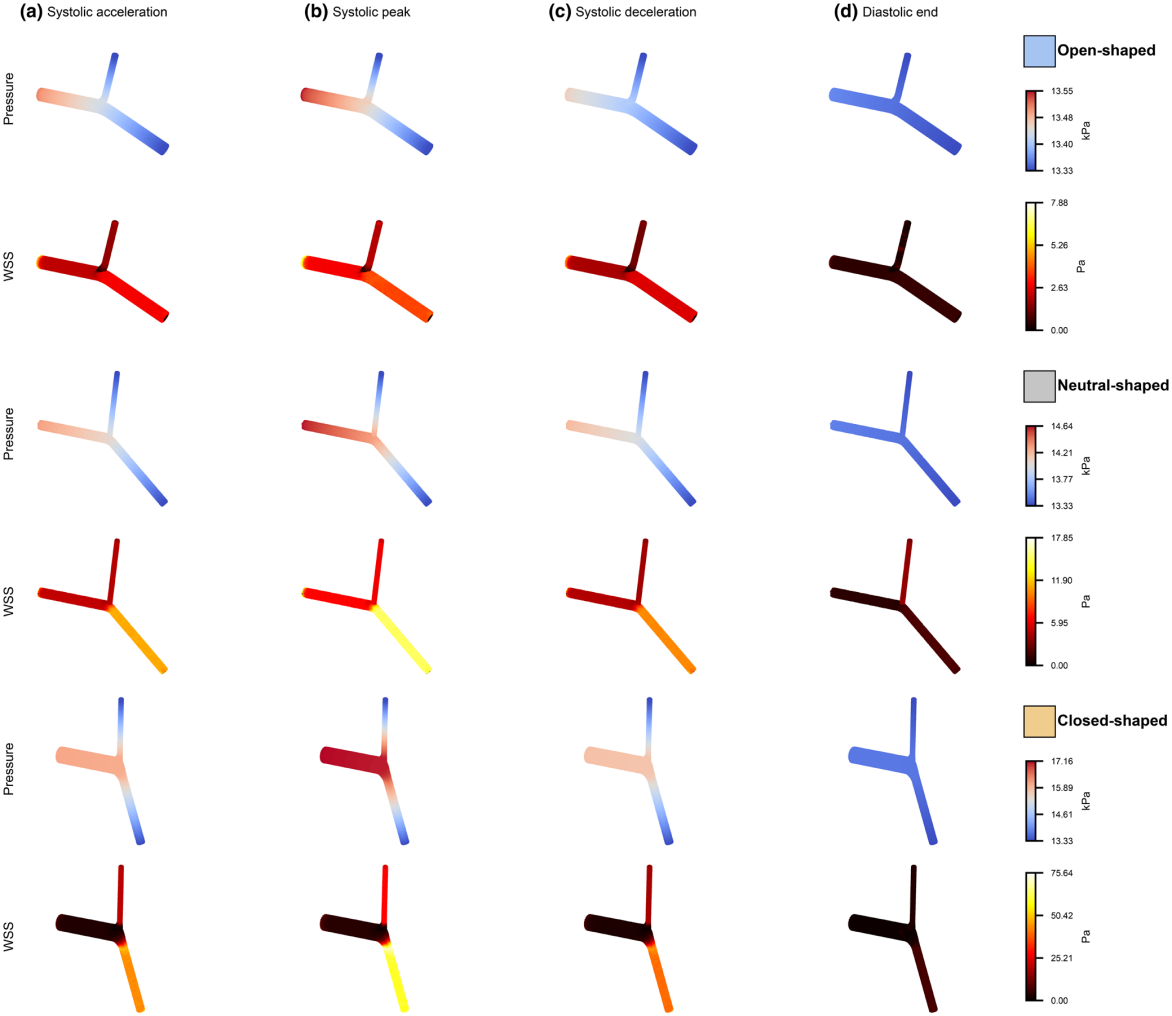
Spatially resolved distributions of pressure (kPa) and wall shear stress (WSS, Pa) are shown for open‐shaped, neutral‐shaped, and closed‐shaped bifurcations at four key phases of the cardiac cycle: (a) systolic acceleration, (b) systolic peak, (c) systolic deceleration, and (d) diastolic end.

Analysis of the hemodynamic parameters within the bifurcation configurations at peak systole (Table 4, Figure 3) revealed differences in the maximum pressure and WSS. Landmark analysis revealed that peak systolic conditions are exemplified by readings taken at 0.09 s (Systolic peak, Landmark B as designated in Figure 1g and visualized in Figure 3b).

**TABLE 4:** Wall shear and pressure parameters within the bifurcation configurations at peak systole.

Parameter	Open‐shaped	Neutral‐shaped	Closed‐shaped
WSS (Pa), Median	1.45	4.74	18.04
WSS (Pa), Max	7.88	17.85	75.64
Max pressure (kPa)	13.55	14.64	17.16

The closed‐shaped configuration demonstrated the highest pressure and WSS metrics during peak systole. The maximum WSS was 75.64 Pa in the closed configuration, which was greater than the values of 17.85 Pa (neutral) and 7.88 Pa (open) (Figure 2a–c). Similarly, the maximum pressure reached 17.16 kPa in the closed configuration, whereas it reached 14.64 kPa in the neutral model and 13.55 kPa in the open geometry.

At the initial measurement time (0.048 s, Systolic acceleration, Landmark A as designated in Figure 1g and visualized in Figure 3a), the median pressure was highest in the closed configuration (14.89 kPa), followed by the neutral (13.78 kPa) and open (13.40 kPa) configurations. The median WSS followed the same trend, with values of 13.04 Pa (closed), 3.51 Pa (neutral), and 1.13 Pa (open).

During the late cardiac cycle phase, characterized by low flow, the median WSS remained low across all three configurations: 0.25 Pa (open), 1.53 Pa (neutral), and 1.98 Pa (closed) (Figure 3d). The pressure maxima at this time point were also close to the minimum pressure values for all three configurations (e.g., Open Max pressure of 13.36 kPa vs. Min pressure of 13.33 kPa).

## 4 DISCUSSION

The results of this study reveal that human cerebral arterial bifurcations are not merely anatomical conduits but also distinct physical regulators of the local inflammatory microenvironment. We identified a fundamental “hemodynamic dichotomy” dictated by vascular geometry: the prevalent open‐shaped configuration (87.4%) functions as a low‐energy, high‐stagnation zone, whereas the rare closed‐shaped configuration (2.2%) acts as a high‐energy, mechanical stress concentrator (Figure 1c). This finding suggests that susceptibility to vascular inflammation is not random but rather physically encoded in the branching architecture of the arterial tree, a finding that aligns with the increasing recognition of hemodynamic factors in cerebrovascular disease pathogenesis.

The open‐shaped bifurcation, despite being the canonical “efficient” geometry described by Murray's law, presents a significant biological vulnerability: flow stagnation. Our simulations show that this configuration results in the highest RRT (median: 1.68 s), which is more than 12 times longer than that of the closed configuration. Biologically, high RRT is a critical physical determinant of immune cell recruitment (Nowicki et al., 2014). A prolonged residence time physically increases the contact duration between circulating monocytes and the endothelial surface, exponentially increasing the probability of adhesion and thrombus deposition (Jiang et al., 2025; Riccardello et al., 2018).

Furthermore, the open configuration yielded the highest ECAP (0.092). High ECAP, driven by low time‐averaged wall shear stress and directional oscillation, is a known physical agonist of endothelial dysfunction (Dake et al., 2024; Ramses & Agu, 2025; Wang et al., 2021). In these low‐shear regions, the lack of laminar suppression allows for the upregulation of proinflammatory adhesion molecules. Previous studies have confirmed that the plaque burden, even in non‐stenotic arteries, is significantly associated with these regions of high ECAP and RRT (Lan et al., 2024). Thus, the open‐shaped geometry, while energetically favorable for bulk flow, creates a “physically primed” microenvironment for atherosclerosis by fostering stagnation and lipid accumulation at the lateral walls. This computational prediction is strongly supported by recent histopathological evidence demonstrating that larger vessels within the Circle of Willis exhibit significantly greater percentages of arterial calcification compared to smaller vessels (Akhavan et al., 2026).

In sharp contrast, the closed‐shaped configuration generates a markedly different physical environment. These bifurcations are characterized by extreme mechanical forces, with the median TAWSS reaching 7.4 Pa and the peak systolic WSS exceeding 75 Pa. While this high‐velocity “nozzle effect” eliminates flow stagnation—preventing plaque accumulation—it introduces the risk of direct mechanical inflammation (Kandangwa et al., 2025).

Shear stresses of this magnitude are supraphysiological; they can trigger endothelial erosion or structural fatigue (Dolan et al., 2013), and activate mechanically activated ion channels (Coste et al., 2010; Ranade et al., 2014). Greater hemodynamic stresses at bifurcations have been explicitly linked to the initiation of aneurysms (Boniforti et al., 2022; Ćmiel‐Smorzyk et al., 2022; Di Achille et al., 2014; Guo et al., 2023; Himburg et al., 2004; Rayz et al., 2010). High pressure gradients observed in closed bifurcations (max pressure >17 kPa) also increase the transmural stress. While this geometry creates a local stress concentration, we hypothesize that it may play a systemic protective role. The impedance mismatch created by “closed” narrowing generates wave reflections that dampen pulsatile energy, potentially shielding distal capillary beds from barotrauma (Chen et al., 2008). However, this protection comes at the cost of local endothelial trauma at the bifurcation apex (Meng et al., 2007; Wang et al., 2014).

Furthermore, shear stress‐induced nitric oxide (NO) synthesis plays a significant role. Under physiological conditions, healthy laminar shear stress stimulates endothelial NO synthase (eNOS), producing NO to maintain a quiescent, anti‐inflammatory vascular phenotype (Kopych et al., 2025). In the dominant open‐shaped configurations, the observed low time‐averaged wall shear stress combined with high ECAP likely blunts eNOS activity, severely reducing local NO bioavailability (Dhawan et al., 2010; Hasan et al., 2025). This localized NO deficiency removes a critical atheroprotective mechanism, providing a permissive environment for the upregulation of proinflammatory adhesion molecules and focal lipid accumulation. Conversely, while the closed‐shaped configuration eliminates flow stagnation, the shear stresses it generates may drive NO dysregulation or cause direct mechanical stripping of the endothelium (Metaxa et al., 2008; Wang et al., 2025).

Historically, the study of vascular architecture has been dominated by the search for optimality. Since the seminal work of Cecil D. Murray in 1926, the guiding principle of vascular biology has been the “law of minimum work,” which posits that biological systems evolve to minimize the metabolic cost of operation (Murray, 1926b). In the context of arterial branching, Murray's law predicts a specific geometric relationship between the parent vessel and its daughter branches ($D^{3}=\sum d^{3}$), a configuration that theoretically minimizes the energy required to maintain blood volume and overcome viscous drag. This “cubic law,” alongside the “square law” or area law ($D^{2}=\sum d^{2}$), which preserves flow velocity, suggests that arterial bifurcations should fundamentally act as impedance‐matching junctions that facilitate the smooth, energy‐efficient transmission of blood.

Under these standard interpretations, the canonical arterial bifurcation is open‐shaped. In this configuration, the diameter of the parent vessel (D) is less than the sum of the diameters of the daughter vessels ($d_{\max}+d_{\min}$), ensuring that the total cross‐sectional area expands or remains constant across the junction. This geometry prevents flow acceleration, minimizes energy dissipation, and maintains the WSS within a narrow, homeostatic range.

However, in our study, we identified the persistent presence of a closed‐shaped bifurcation defined by the inequality $D>d_{\max}+d_{\min}$; this configuration describes a vascular junction where the parent vessel is wider than the combined width of its downstream branches. Geometrically, this represents a convergence—a narrowing of the vascular lumen that ostensibly contradicts the principles of optimal transport. Instead of expanding the flow channel to reduce resistance, the closed bifurcation constricts it, creating a “nozzle” effect that should theoretically increase resistance, accelerate flow, and dissipate energy.

The existence of such a configuration, particularly in the delicate vasculature of the brain and arterial and venous spleen (Dadashev, Miltykh, et al., 2024; Dadashev, Zenin, et al., 2024), presents a significant hydrodynamic paradox. If the vascular system is optimized for energy conservation, why would it retain a geometry that appears designed for energy loss? Is the closed bifurcation a true physiological feature—a specialized regulatory component of the vascular tree—or is it a methodological artifact born from the complexities of postmortem corrosion casting?

Studies utilizing CFD on cerebral arteries with varying degrees of stenosis (which geometrically mimics the “closed” branch point) confirm that decreasing the area ratio (making the vessel more “closed”) leads to an exponential increase in the pressure drop. In a study of cerebral artery stenosis, a reduction in the contraction area ratio dD was directly correlated with increased pressure loss and flow energy dissipation (Rahma et al., 2022).

The drastic reduction in the cross‐sectional area characteristic of closed‐shaped bifurcations creates a significant impedance mismatch, resulting in positive wave reflections. While this mechanism increases the mechanical load on the parent vessel, we suppose it could act as a functional protective barrier that attenuates pulse pressure transmission, which may theoretically shield distal microvessels from potential barotrauma. This aligns with clinical observations that wave reflections from high‐resistance peripheral beds contribute to central aortic stiffening and hypertension, but locally, they effectively “dampen” the pulsatility entering the organ (Haidar et al., 2021; Zambanini et al., 2005).

However, the observation of a closed‐shaped phenotype could be linked to the corrosion casting technique. This technique involves the injection of a composition of powdered polymethyl methacrylate and a liquid monomer (e.g., methyl methacrylate) into the vascular system, its polymerization into a solid plastic replica, and the subsequent maceration of the surrounding tissue. While corrosion casting provides unparalleled three‐dimensional resolution of the vasculature, it is an intrusive chemical process subject to artifacts that can distort geometry. The resins used in vascular casting undergo volumetric shrinkage during the transition from a liquid monomer to a solid polymer. This shrinkage is intrinsic to the formation of covalent bonds, which pack atoms closer together than van der Waals forces in the liquid state. Methyl methacrylate has a reported volumetric shrinkage of approximately 8%–9% (Weiger et al., 1986). While these findings seem minor, empirical measurements of rat aortas have revealed that the diameter decreases by 19.6%, affecting the curvature and branching angles (Shih et al., 2022). The polymerization of methacrylates is exothermic. The heat generated is proportional to the volume of resin, whereas heat dissipation occurs through the surface area. A large parent vessel (D) has a higher volume‐to‐surface ratio than small daughter vessels do (d). Consequently, the core of the parent cast may reach higher peak temperatures than the daughters do. Compared with cooler daughter vessels, higher temperatures can alter the polymerization kinetics, potentially causing the parent cast to shrink differently or expand the vessel wall (thermal relaxation) before setting. If the parent vessel volume is large enough to sustain a “liquid core” while the daughters set rapidly, the parent might retain its dimensions better (or shrink later, pulling away from the wall), thereby creating a mismatch in the parent–daughter diameter ratios.

Morphological evidence supports the existence of closed‐shaped bifurcations through the presence of intra‐arterial cushion, which creates functional narrowing at the daughter vessel origin. This structural mechanism is conserved across diverse vascular beds and functions as a regulator in the kidney (glomerular filtration) (Kopitkó et al., 2022), brain (protecting parenchymal microcirculation) (Harrison et al., 2002; Sangiorgi et al., 2018), and eye (retinal flow) (Ninomiya et al., 2005). The widespread presence of these cushions confirms that the observed diameter reduction is a deliberate anatomical strategy for flow control rather than a measurement error.

However, the fact that these “constrictions” appear at consistent anatomical locations (e.g., renal, heart, and splenic vasculature) at specific vasculature division levels (Figure 1f) rather than randomly suggests that the casting process is amplifying a real anatomical feature rather than creating a structure de novo. The “closed” bifurcation is the functional choke point of the arterial tree. While the closed geometry increases the local stress (sacrificing the parent vessel), it may function as a low‐pass filter, damping pulsatile energy before it reaches the delicate downstream capillary beds. Although corrosion casts may exaggerate their degree of damage due to shrinkage and spasm, we propose the hypothesis that the underlying geometry might represent an anatomical adaptation to help protect the microcirculation from the pulsatile environment of the macrovasculature.

Building upon the hypothesis that closed‐shaped bifurcations serve a systemic protective role by dampening pulsatile energy, it is highly likely that this architectural distribution is not static across a lifespan. We suppose that if these geometries serve as a structural barrier shielding the distal microcirculation, their prevalence might differ between healthy young adults and older populations. It is theoretically possible that the sustained high mechanical stress localized at these focal points could gradually induce structural fatigue and maladaptive remodeling over time. If such remodeling were to occur, the degradation of this geometric damping effect could conceivably contribute to the excessive penetration of pulsatile energy into downstream microvessels. However, without age‐stratified or longitudinal data, this concept remains strictly a hypothesis. Future morphometric studies are necessary to determine whether aging and cardiovascular risk factors actually influence the distribution and structural integrity of these bifurcation configurations.

### 4.1 Limitations

Limitations include the use of rigid‐wall assumptions and the analysis of single representative geometries rather than full‐ensemble simulations. While rigid‐wall models have been validated for small vessels, future work incorporating fluid–structure interactions and subject‐specific inlet conditions may refine the hemodynamic signatures identified here. For example, closed‐shaped bifurcations show pressure spikes. In a living vessel with elastic walls, the vessel distends to relieve this pressure. The rigid wall assumption in high‐pressure scenarios likely overestimates stress. Consequently, the rigid wall assumption forces all fluid energy to be dissipated as shear and pressure, likely overestimating the absolute magnitude of the stress. This is particularly relevant for the closed‐shaped configuration, which acts as a focal geometric constriction and subsequently exhibited the highest hemodynamic loads, such as a maximum WSS of 75.64 Pa. While the absolute values in the closed configuration could be increased due to the rigid boundaries, the relative pathophysiological interpretation remains: closed geometries inherently concentrate mechanical stress and dissipate energy, whereas open geometries promote flow stagnation. Future studies utilizing fluid–structure interactions are necessary to determine the precise in vivo magnitudes of these geometric stress concentrators.

Another limitation of the current study is the reliance on a single, normotensive static outlet pressure (100 mmHg) to define the boundary conditions. This approach was intentionally chosen to conduct a controlled, qualitative comparison of the open, neutral, and closed configurations under identical baseline conditions, strictly isolating the effect of bifurcation geometry on the local microenvironment. However, hypertension is one of the most common comorbidities observed in patients with cerebrovascular events. Building upon the foundational geometric profiles established here, future multiscale studies may incorporate variable pressure arrays and temporal analyses to model hypertensive pathophysiology and its specific impact on these distinct bifurcation architectures. Consequently, the absolute values of WSS and pressure reported should be interpreted with caution; they represent idealized physical magnitudes rather than precise in vivo patient measurements. The value of these simulations lies in the relative, qualitative differences observed between the geometries, demonstrating how these “building blocks” inherently govern local fluid dynamics.

On the other hand, this study utilizes corrosion casting as a primary method of data collection. Compared with those in in vivo pressurized states, cast resins can undergo shrinkage (polymerization shrinkage), and vessels lose tone after death, which could affect morphometric data. This shrinkage is known to affect vascular curvature and distort natural branching angles. Consequently, the angles preserved in the casts may not accurately reflect the true in vivo geometry. To prevent these casting artifacts from confounding our results, we used theoretical angles to create a standardized geometric baseline. Biological in vivo branching angles naturally deviate from theoretical ideals due to surrounding tissue constraints and structural remodeling. Such deviations can independently influence local flow separation, secondary flow patterns, and WSS distribution. Future studies utilizing high‐resolution in vivo imaging (such as 4D flow MRI) are required to investigate how branching angles interact with these configurations.

## 5 CONCLUSIONS

This study demonstrated that cerebral arterial bifurcations generate distinct biomechanical profiles that may predispose specific sites to pathological remodeling. The dominant open‐shaped geometry physically predisposes the endothelium to stagnation‐induced activation (high ECAP), offering a mechanistic explanation for the focal nature of intracranial atherosclerosis. Conversely, closed‐shaped geometries subject the vessel wall to mechanical trauma (high WSS). These findings highlight the critical role of vascular in dictating the local physical forces that are known drivers of mechanobiological signaling, bridging the gap between gross anatomy and localized vascular vulnerability.

By establishing quantitative hemodynamic profiles for each bifurcation type, this study provides a foundational framework for future multiscale models of cerebral blood flow. We hypothesize that the translational value of these findings could be explored through the future clinical application of standardized morphometric indices, specifically the length‐to‐radius ratio ($FF_{1}$), asymmetry ratio (γ), and area ratio (η). If these geometric parameters can be reliably measured using modern noninvasive imaging modalities like high‐resolution CT and MRI, they could potentially serve as objective criteria for defining optimal or normal cerebral vascular architecture in vivo. These results may assist in identifying vascular regions at greater mechanical or biological risk, improving computational models of disease progression, and informing the design of targeted diagnostic or therapeutic strategies. Furthermore, this bifurcation‐centered approach underscores the importance of studying the cerebral vasculature not only at the level of major arteries but also through the cumulative behavior of its smallest architectural “building blocks.”

## AUTHOR CONTRIBUTIONS

**Ilias Miltiadis:** Conceptualization; formal analysis; investigation; methodology; software; visualization. **Oleg Zenin:** Conceptualization; methodology; project administration; supervision. **Edgar Kafarov:** Methodology; resources. **Mahsa Alavi:** Formal analysis; investigation; validation. **Nadezhda Stashevskaya:** Formal analysis; investigation; validation. **Pavel Burko:** Conceptualization; methodology; project administration; supervision.

## FUNDING INFORMATION

The authors did not receive support from any organization for the submitted work.

## CONFLICT OF INTEREST STATEMENT

The authors have no relevant financial or nonfinancial interests to disclose.

## ETHICS STATEMENT

The study was conducted in accordance with the Declaration of Helsinki, and approved by the Ethics Committee of A.A. Kadyrov Chechen State University (protocol code 490/41‐33, December 15, 2025).

## Data Availability

The data that support the findings of this study are available from the corresponding author upon reasonable request.

## APPENDIX A

### A.1 GOVERNING EQUATIONS

Blood was modeled as an incompressible fluid governed by the continuity equation:

(A.1)
∇·u=0

And the Navier–Stokes momentum equation:

(A.2)
$$\rho \left(\frac{\partial u}{\partial t}+\left(u\cdot \nabla \right)u\right)=-\nabla p+\nabla \cdot \tau$$

Blood rheology was defined by the Carreau non‐Newtonian model:

(A.3)
$$\mu =\mu_{\infty }+\left(\mu_{0}-\mu_{\infty }\right){\left[1+{\left(\lambda \dot{\gamma }\right)}^{2}\right]}^{\frac{n-1}{2}}$$

where $\mu_{0}=0.056\;\mathrm{Pa}\cdot s$ is the dynamic viscosity under zero shear stress conditions and where $\mu_{\infty }=0.0035\;\mathrm{Pa}\cdot s$ is the dynamic viscosity under maximum shear stress conditions, λ=3.313; n=0.3568.

### A.2 BRANCHING EQUATIONS

The values of the branching angles ($\alpha_{\max}$ and $\alpha_{\min}$) were calculated via the equations of Murray C. D.:

(A.4)
$$\alpha_{\max}=\arccos\left(\frac{D^{4}+{d_{\max}}^{4}-{\left(D^{3}-{d_{\max}}^{3}\right)}^{\frac{4}{3}}}{2D^{2}{d_{\max}}^{2}}\right)$$

(A.5)
$$\alpha_{\min}=\arccos\left(\;\frac{D^{4}+{d_{\min}}^{4}-{\left(D^{3}-{d_{\min}}^{3}\right)}^{\frac{4}{3}}}{2D^{2}{d_{\min}}^{2}}\right)$$

### A.3 HEMODYNAMIC METRICS

Wall Shear Stress (WSS):

(A.6)
$$\tau_{w}=\mu \frac{\partial u_{t}}{\partial n}$$

where $u_{t}$ is the tangential velocity vector and n represents the direction normal to the vessel wall.

Time‐Averaged Wall Shear Stress (TAWSS):

(A.7)
$$\text{TAWSS}=\frac{1}{T}\int_{0}^{T}\left|\vec{\tau_{w}}\right|\mathrm{dt}$$

Transverse WSS (transWSS):

(A.8)
$$\text{transWSS}=\frac{1}{T}\int_{0}^{T}\left|\vec{\tau_{w}}\cdot \left(\vec{n}\times \frac{\int_{0}^{T}\vec{\tau_{w}}\;\mathrm{dt}}{\left|\int_{0}^{T}\vec{\tau_{w}}\;\mathrm{dt}\right|}\right)\right|\mathrm{dt}$$

Oscillatory Shear Index (OSI):

(A.9)
$$\mathrm{OSI}=\frac{1}{2}\left(1-\frac{\left|\frac{1}{T}\int_{0}^{T}\vec{\tau_{w}}\;\mathrm{dt}\right|}{\text{TAWSS}}\right)$$

Endothelial Cell Activation Potential (ECAP):

(A.10)
$$\text{ECAP}=\frac{\mathrm{OSI}}{\text{TAWSS}}$$

Relative Residence Time (RRT):

(A.11)
$$\mathrm{RRT}=\frac{1}{\left(1-2\cdot \mathrm{OSI}\right)\cdot \text{TAWSS}}$$
